# A Retrospective Pre-Post Observational Study Of The Effectiveness of Cognitive Stimulation Therapy and Reality Orientation & Reminiscence Therapy in Older Chinese People with Dementia

**DOI:** 10.1007/s10823-025-09548-7

**Published:** 2025-11-20

**Authors:** Frank Ho-yin Lai, Kathy Ka-ying Yu, Eddie Yip-kuen Hai, Ben Chi-bun Yip, Catherine Kam-fung Chan, Georg S. Kranz

**Affiliations:** 1 Northumbria University, Newcastle upon Tyne, United Kingdom; 2 Mental Health Research Centre, The Hong Kong Polytechnic University, Hong Kong, Hong Kong, Hong Kong; 3 The Salvation Army Tai Po Integrated Service for Senior Citizens, The Salvation Army China, Hong Kong, Hong Kong, Hong Kong; 4 University of Sunderland, Sunderland, United Kingdom; 5 Tung Wah College, Hong Kong, China; 6 Newcastle upon Tyne Hospitals NHS Foundation Trust, Newcastle upon Tyne, United Kingdom; 7 Hong Kong Polytechnic University, Hong Kong, China

**Keywords:** Cognitive stimulation therapy, Executive functions, Attention, Dementia, Activities of daily living

## Abstract

Cognitive deficits such as attentional impairment and executive dysfunction significantly impact daily living activities in older adults with dementia. This study aimed to evaluate the efficacy of Cognitive Stimulation Therapy (CST) compared to Reality Orientation & Reminiscence Therapy (RO&RM) in improving attention, episodic memory, and executive functions in older Chinese adults with mild to moderate dementia. Additionally, it sought to explore the relationship between attention improvements and changes in executive functions. A retrospective observational pre–post study was conducted from September 2018 to July 2021, involving 160 participants aged 65 or above, diagnosed with dementia. Participants were divided into CST (n = 80) and RO&RM (n = 80) groups, receiving six weeks of daily 1-h sessions. Attention and episodic memory were assessed using the Kendrick Cognitive Test for the Elderly (KCTE), and executive functions were evaluated using the Chinese Disability Assessment for Dementia (CDAD). CST significantly improved attention (*p* = 0.002) and episodic memory (*p* = 0.010), with attention improvements being more pronounced. RO&RM showed no significant improvement in these areas. Overall, executive functions did not significantly change, but a positive correlation was found between improved attention and reduced decline in executive functions. CST demonstrated notable potential in enhancing attentional capacities and episodic memory in older Chinese adults with dementia. However, its impact on executive functions was inconsistent. Future research should involve larger sample sizes, longer follow-up periods, and explore combining CST with other therapies to maximise therapeutic potential. This study underscores the importance of culturally adapting CST to better fit the needs of the Chinese dementia population.

## Introduction

Today, about 50 million people worldwide live with dementia, a number that is estimated to rise to 75 million cases by 2030 (World Health, 2018). Due to their progressive deterioration in cognitive function, older adults with dementia are significantly more dependent on help when performing activities of daily living (ADL).

Attentional impairment, executive dysfunction, and difficulties with ADLs are interconnected in older adults with mild to moderate dementia (Rabinovici et al., 2015; Swanberg et al., 2004). The inability to filter information from external sources and to focus on a particular task is a common symptom in dementia (Ashizawa et al., 2021). Although functionally separate from memory, problems with attention can also impact a person’s ability to learn or recall information (Bondi et al., 2017). Attention represents a fundamental element of executive function. The term executive functions refers to advanced cognitive skills that require planning, coordinating, solving issues, and regulating oneself (Kudlicka et al., 2011). Current evidence suggests that attention may be the first non-memory domain to be affected in Alzheimer’s disease (AD), the most common cause of dementia (Malhotra, 2018; Nasiri et al., 2023), before the occurrence of deficits in language and visuospatial functions (Schneider, 2017). Individuals who struggle to sustain focus on a particular task may encounter incomplete or inadequately executed plans (Bondi et al., 2017). This is consistent with the hypothesis that difficulties with ADL, which occur in even mildly demented patients, are related to attentional deficits (Posner et al., 2006; Veríssimo et al., 2022). Furthermore, their difficulties in multitasking (e.g., engaging in cooking while conversing) may obstruct complex activities necessitating the coordination of multiple sequential steps (Posner et al., 2006).

Attentional impairment and executive function (EF) exhibit a profound interrelation, as attention serves as a fundamental element underpinning numerous higher-order cognitive processes categorised within the domain of EF (Baddeley, 2003; Ma et al., 2014). The constructs of EF are intricately associated with attentional mechanisms, and a plethora of neuropsychological assessments that claim to evaluate EF are frequently employed interchangeably as measures of attention (Draheim et al., 2022; McCabe et al., 2010). Disruptions in attentional capacities adversely impact EF by undermining the ability to concentrate, transition, and sustain cognitive efforts. This correlation is particularly pronounced in individuals diagnosed with dementia. Clinical evaluations indicate that individuals with dementia frequently encounter substantial difficulties in executing ADLs, even when formal assessments of non-memory cognitive functions, such as language, praxis, and visuospatial skills, reveal minimal or no impairments (Carlsson et al., 2009). As a result, the compromised capacity to filter out distractions and maintain sustained focus on tasks contributes to an increased reliance on caregiving support. These observations have prompted hypotheses suggesting that individuals with dementia may exhibit attentional deficits, which may manifest as an early symptom of their condition (Sung et al., 2023a, 2023b). For example, older adults with mild to moderate dementia may experience challenges in selective attention, such as difficulties in managing financial affairs due to being sidetracked by extraneous details or interruptions (Cubelli et al., 2023). Shortcomings in this aspect may produce serious hurdles in sticking to instructions, tackling problems, or incorporating new insights (Baddeley, 2021; Jefferson et al., 2006). For instance, individuals may inadvertently forget the sequential steps of a recipe or lose track of their current activities. Their diminished cognitive flexibility impedes their ability to transition smoothly from one task to another, such as shifting from culinary activities to cleaning tasks.

Addressing these cognitive deficits through specifically designed interventions can facilitate the preservation of autonomy and enhance overall quality of life. Psychosocial approaches like Reality Orientation (RO), Reminiscence Therapy (RM), and Cognitive Stimulation Therapy (CST) are mainly directed towards individuals suffering from cognitive issues, notably those with dementia. While they share some similarities, they differ in their approaches, goals, and techniques.

RO represents a therapeutic modality that seeks to mitigate confusion and disorientation in individuals afflicted with dementia by bolstering their awareness of temporal, spatial, and personal contexts (Camargo et al., 2015). This intervention concentrates on diminishing disorientation by reinforcing present awareness. RO encompasses the provision of consistent and repetitive information regarding the surrounding environment, including details such as the date, time, location, and the identities of individuals present (Li et al., 2023). RO interventions has been beneficial in aiding individuals with cognitive deficits to maintain attention on relevant tasks, thereby improving their orientation and recall abilities (Draheim et al., 2022; Mathew, 1987). RO is associated with reduced violence and aggression, which may be linked to improved attention and cognitive control (Patton, 2003). Through the implementation of orientation boards, calendars, clocks, and verbal reminders, RO endeavors to anchor individuals in reality while alleviating confusion (Wang et al., 2022). Clinical research has demonstrated that RO can provide meaningful enhancements in cognitive and behavioral performance for patients experiencing dementia (Spector et al., 2000). While beneficial for certain individuals, RO may provoke frustration or distress among those with advanced dementia if it is excessively employed or applied without sensitivity (Li et al., 2023).

RM involves the active recall of past experiences, which requires cognitive engagement and can stimulate attention. This process can help maintain or improve cognitive functions, including attention, especially in older adults (Cammisuli et al., 2022). RM entails stimulating individuals to recollect and articulate past experiences, frequently from their formative years, as a means to foster emotional well-being and enhance self-esteem (Woods et al., 2005). This approach involves the retrieval of past experiences to bolster psychological health. RM prioritizes personal memories and life narratives, often employing prompts such as photographs, music, or familiar objects to amplify emotional wellness, cultivate a sense of identity, and improve mood. Nonetheless, the efficacy of RM can exhibit considerable variability contingent upon the individual's cognitive capacities, personality traits, and readiness to participate in the process (Woods et al., 2005). Fundamentally, the act of recalling past experiences may occasionally elicit painful or traumatic memories, resulting in emotional distress or agitation (Korte et al., 2012; Subramaniam & Woods, 2012). Moreover, critics contend that RM may place excessive emphasis on past experiences, potentially overlooking opportunities to engage individuals in meaningful activities in the present (Subramaniam & Woods, 2012).

Cognitive Stimulation Therapy (CST) serves as a thoroughly planned, research-backed group strategy that seeks to advance cognitive performance (Reshmathi et al., 2024), nurture psycho-social well-being (Gonzalez-Moreno et al., 2022), and raise the living standards of individuals with mild to moderate dementia (Desai et al., 2024). This therapeutic method invites participants to take part in various tasks and dialogues focused on boosting cognitive skills including thought processes, recollection, and problem-solving abilities. The activity sessions are typically characterized by structured exercises that not only aim to invigorate cognitive faculties but also encourage social engagement and interaction (Foldi et al., 2002). CST is acknowledged as a method grounded in evidence that does not rely on medication, showing effectiveness in enhancing various cognitive areas such as attention, memory, and executive functions while also positively impacting the quality of life and social connections for those experiencing mild to moderate dementia (Zucchella et al., 2018). Empirical studies have substantiated that CST can result in cognitive enhancements and improvements in quality of life for individuals within the specified dementia severity spectrum (Woods et al., 2012). Research has particularly indicated that CST may bring about notable advancements in memory, attention, and executive functions, based on neuropsychological assessments (Gonzalez-Moreno et al., 2022; Reshmathi et al., 2024). The group format of CST provides a structured environment where participants engage in activities that require focused attention. This setting can help improve attentional control by encouraging participants to concentrate on tasks and interact with others (Knowles, 2010). Furthermore, a comprehensive meta-analysis of CST protocols has revealed considerable benefits in global cognition, which encompasses executive functions, language capabilities, and working memory. These cognitive improvements were consistently observed across various trials, underscoring the robustness of CST's influence on cognitive health (Desai et al., 2024). A systematic review and meta-analysis have indicated that CST can enhance specific dimensions of executive functions, such as inhibitory control and working memory, as evaluated through targeted tasks like the Stroop and Corsi Block tasks. Another meta-analysis has illustrated significant training effects for tasks that were explicitly targeted by the intervention, alongside smaller effects for near-transfer and far-transfer tasks, thereby indicating a degree of generalization of benefits (Nguyen et al., 2019). Moreover, CST has been correlated with reductions in neuropsychiatric symptoms and improvements in communication skills, which are vital for sustaining social interactions and facilitating daily functioning (Desai et al., 2024). By fostering focus, problem-solving abilities, and social engagement, CST contributes to enhancements in attention (Desai et al., 2024). Additionally, CST sessions frequently incorporate activities that specifically target working memory and language skills, both of which are intricately connected to attention (Spector et al., 2000). Documented improvements in these cognitive domains suggest that CST possesses the potential to augment cognitive processes that underpin attention.

Episodic memory tasks can be tailored to resonate with participants’ lived experiences, enhancing both engagement and ecological validity (Wong et al., 2018). Episodic memory is closely tied to everyday functioning. Difficulties in remembering recent conversations, appointments, or where items are placed can significantly impair independence and quality of life. Improvements in episodic memory may therefore translate into better real-world outcomes (Albert et al., 2013). Episodic memory has been shown to be responsive to non-pharmacological interventions, including CST. It is a practical and meaningful outcome to assess the efficacy of such therapies, especially when pharmacological options are limited or have modest effects (Spector et al., 2003).

Taken together, accumulating evidence indicates that problems with attention are a significant factor contributing to difficulties in ADL in mild to moderate dementia. However, how attention affects EF and thereby ADL performance, and whether CST can increase functional performance by increasing attention, is yet to be determined. The rationale of this study was to compare the efficacy of CST and RO&RM in supporting these cognitive and functional domains by documenting the change of attention, episodic memory and executive functions in older adults with mild to moderate dementia after the CST, additionally, to study if there was any association between improved attention and changes in executive functions. We hypothesised improved attention and episodic memory after CST (hypothesis 1). We further hypothesised that daily CST compared to a control training consisting of a combination of RO and RM training, improves EF (hypothesis 2). Finally, we hypothesised that the improvement in attention after CST is related to the increase in EF (hypothesis 3). More specifically, we hypothesised that improved attention drives improvement in EF (hypothesis 4).

## Methods

### Study Design and Treatment Protocol

We conducted a retrospective observational pre–post study. Through data retrieval from the database, this retrospective study investigates the efficacy of Cognitive Stimulation Therapy (CST) compared to Reality Orientation and Reminiscence Therapy (RO&RM) in older adults with mild to moderate dementia in the Chinese population. Data of one hundred and sixty participants, between September 2018 and July 2021, who received six weeks of daily 1-h sessions of into CST group (*n* = 80) and RO&RM group (*n* = 80) were retrieved and grouped according to the last digit of their identity document, with odd number to the CST group and even number to the RO&RM group.

Based on the protocol as suggested by Spector et al. (2003), the CST in this study provided a structured, evidence-based approach to cognitive stimulation for individuals with dementia (Spector et al., 2003). Its emphasis on engaging activities, social interaction, and person-centered care makes it a valuable intervention for improving cognitive function and quality of life. The CST group, conducted by a trained therapy assistant who were aware of the focus of the study, consisted of six weeks of daily 1-h sessions. Due to staffing and programme management considerations, the structure of CST used in current study was different from the original protocol proposed 14 sessions of structured 45-min to 1-h group therapy sessions normally conducted over 7 weeks.

To ensure cultural relevance and participant engagement, the standard CST protocol was adapted for the Chinese population in several key ways, drawing on established frameworks for cultural adaptation (Wong et al., 2018). First, the content of CST sessions was localized by incorporating culturally familiar materials and themes. For example, instead of using Western holidays or foods in discussion activities, sessions included references to Chinese festivals (e.g., Mid-Autumn Festival, Lunar New Year), traditional foods (e.g., dumplings, mooncakes), and local customs. Music and visual materials were also adapted to include traditional Chinese songs and imagery that resonated with participants’ lived experiences. Second, the structure of group interactions was modified to reflect the collectivist and often more reserved communication style typical in Chinese culture. Facilitators were trained to encourage participation through indirect prompts and group-based activities rather than relying heavily on individual verbal expression. Activities such as storytelling, calligraphy, and tea appreciation were introduced to promote engagement in a culturally respectful and familiar manner. Finally, language and facilitation style were adjusted to ensure clarity and comfort. All sessions were conducted in Cantonese or Mandarin, depending on the group, and facilitators were trained to use culturally appropriate metaphors and examples. These adaptations were informed by formative research and pilot testing, which highlighted the importance of tailoring CST to the sociocultural context to maximize its acceptability and effectiveness.

Participants in the RO&RM arm had six weeks of daily 1-h sessions. Another trained therapy assistant, who without any involvement of the CST training and planning, conducted the RO group training with the use of orientation boards, calendars, clocks, verbal reminders, and different environmental cues to reduce participants’ confusion, disorientation and behavioral problems. In RM contents, the therapy assistant adopted psychosocial strategies, such as storytelling, looking at old photos, listening to music from the past, and discussing significant life events to stimulate memory and personal identity. Participants were encouraged the group to recall and share past events using old pictures and objects.

### Study Participants

Data collected are participants from a day activity centre for people with mild-to-moderate dementia between September 2018 to July 2021. For study inclusion, participants had to be 65 years old or above, live in Hong Kong and had a diagnosis for dementia in medical history and had to meet ICD-10 criteria for dementia, diagnosed by a psychiatrist. Furthermore, participants had to have cognitive impairment, as determined by the Montreal Cognitive Assessment (MoCA) (Wong et al., 2009), cut-off score ≤ 19. Exclusion criteria were any major neurological illness other than dementia, any psychiatric disorder or a known history of substance abuse. Participants were assessed after informed consent was obtained in the presence of his/her first-degree relatives. Ethics approval was given by the local Research Ethics Committee, and the study was conducted according to the Declaration of Helsinki of 1975.

### Outcome Measures

All participants’ attention and episodic memory were quantified using the Kendrick Cognitive Test for the Elderly (KCTE). The KCTE has been shown to have a specificity of 100% and a sensitivity of 39% when used to diagnose dementia (Baddeley et al., 2001; Kline, 2013; Rai et al., 1991). KCTE has demonstrated reliability and validity across a broad spectrum of older adults, including normal, depressed, and dementing individuals (Burt et al., 1995; Kendrick et al., 1979). The KCTE has been used to evaluate attention and episodic memory in dementia previously (Nurk et al., 2009, 2010). The KCTE, when used alongside the synonym section of the Mill Hill vocabulary scale, has demonstrated sensitivity to cognitive changes over a six-week period (Fish et al., 1986; Gibson et al., 1980). To evaluate our first hypothesis, the KCTE test consists of two components, an Object Learning Test (KOLT) that measures episodic memory of everyday objects, and a Digit Copying Test (KDCT) that quantifies attention and processing speed (Burt et al., 1995; Desrosiers, 1992). This measure is used to determine attention and episodic memory and to monitor these cognitive domains.

The Chinese Disability Assessment for Dementia (CDAD) is a robust and culturally relevant tool for assessing executive function in older adults with dementia. Its focus on initiating, planning, and executing activities provides a practical and ecologically valid measure of executive dysfunction, aligning closely with the real-world challenges faced by older Chinese people with dementia. To evaluate our second hypothesis, the CDAD measures basic and instrumental activities of daily living and is used to determine suitable interventions and to monitor disease progression (Mok et al., 2005). The CDAD is administered through an interview with the caregiver and assesses three types of activities, including Basic (dressing, hygiene), Instrumental (telephoning, housework) and Leisure (recreation) activities; these activities are assessed according to executive functions, divided into “Initiation” (starting an action), “Planning” (organizing, structuring an action) and “Effective performance” (the ability to complete an action) (Nip et al., 2010). The CDAD showed high internal consistency with a Cronbach's alpha of 0.91 and excellent test–retest reliability with an intraclass correlation coefficient (ICC) of 0.99. Additionally, the CDAD was validated against several instruments to establish its construct validity. It showed a high negative correlation with the Global Deterioration Scale (GDS), with a Spearman's rho of −0.89, indicating that as dementia severity increases, functional ability decreases. Overall, the CDAD is a reliable and valid tool for assessing functional disabilities in Chinese older adults with AD, capturing both basic and instrumental activities of daily living while considering cultural nuances.

Data was captured through progress notes before treatment started (as baseline) and after 6 weeks of treatment (post-intervention).

### Statistical Analysis

Data was analyzed using IBM SPSS Statistics version 24 for Windows. Visual inspection and Kolmogorov Smirnov test revealed a non-normal distribution of all measured outcome variables. To evaluate the improved attention and episodic memory after CST, a Wilcoxon Signed Rank Test was conducted to test the effects of treatment on KDCT and KOLT (hypothesis 1). Secondly, the Wilcoxon Signed Rank Test was conducted to test the effects of treatment on EF using CDAD and its nine subscales (EF Initiate, Plan and Effective performance of activities ADL, IADL and Leisure) (hypothesis 2). Followed by a linear regression model to compare KDCT/KOLT change scores between the CST group and RO&RM group. To assess if the improvement in attention after CST is related to the increase in EF, the Wilcoxon Signed Rank Test was further used to compare the treatment effect between the two outcome variables in an exploratory analysis by comparing KDCT and KOLT change scores. We went on to evaluate whether treatment induced changes in CDAD are related to changes in KDCT and KOLT by means of Spearman Correlation (hypothesis 3). This was followed by a conversion of each correlation coefficient to z-scores and by testing the difference between two dependent correlations with one variable in common. Finally, to assess if improved attention drives improvement in EF, the ordinal regression was performed to determine whether changes in CDAD are a function of changes in KDCT (hypothesis 4). The alpha level was set at 0.05 and corrected for multiple comparisons using the Bonferroni procedure.

## Results

### Sample Characteristics

The study included two well-matched groups, CST and RO&RM, with 40 females and 40 males in each group. The demographic characteristics of participants are depicted in Table 1. There was no significant difference between the CST and RO&RM groups regarding age, years of education or cognitive ability at baseline, as determined by MoCA score. Participants in both groups had similar median ages (CST: 78 years; RO&RM: 79 years), years of education (CST: 8 years; RO&RM: 8 years), and baseline cognitive ability as measured by the Montreal Cognitive Assessment, MoCA (CST: 19; RO&RM: 19). Statistical analysis confirmed no significant differences between the groups in age (*p* = 0.163), education (*p* = 0.781), or baseline MoCA scores (*p* = 0.849). The results indicated that the groups were well-matched at baseline, ensuring that any observed differences after training could be attributed to the interventions rather than pre-existing differences, strengthening the study’s internal validity and aligning with methodological standards seen in previous research.

**Table 1:** Sample characteristics

Intervention group	*CST*	*RO & RM*		*P value*
Gender (n; F/M)	40/40	40/40		-
Age	75;78;82	77;79;82		0.163
Years of education	7;8;9	7;8;9		0.781
Baseline MoCA	16.25;19;21	16.25;19;20		0.849

Values indicate the number of subjects (for the variable Gender) and 25th, 50th = median and 75th percentiles (other variables). The p-value corresponds to the comparison of the two groups using Independent Samples Mann–Whitney U Test.

### Attention and Episodic Memory

KCTE total and subscale scores before and after training are depicted in Table 2. Related-Samples Wilcoxon Signed Rank Test revealed a significant improvement in attention, as indicated by a significantly higher KDCT score after CST (*p* = 0.002) but not after RO&RM training (*p* = 0.059). Linear regression analysis confirmed a significant difference between CST and RO&RM using KDCT change scores as dependent variables, corrected for baseline values (*p* = 0.003). In episodic memory, as measured with KOLT, showed an improvement after CST (*p* = 0.010) but not after RO&MT training (*p* = 0.083). However, linear regression analysis showed no significant difference between CST and RO&RM using KOLT change scores as dependent variables, corrected for baseline values (*p* = 0.113). Improvement in attention was significantly stronger than improvement in episodic memory in the CST group, as determined using Related-Samples Wilcoxon Signed Rank Test of KDCT and KOLT change scores (*p* = 0.001).

**Table 2:** Attention and episodic memory before and after training

	*Pre-test*	*Post-test*	*P value*
CST group (*n* = 80)			
KDCT KOLT	72.22 ± 25.76 15.63 ± 6.70	71.62 ± 26.71 15.45 ± 6.85	0.002* 0.010*
RO & RM group (*n* = 80)			
KDCT KOLT	71.38 ± 26.58 15.30 ± 6.69	71.61 ± 26.71 15.45 ± 6.84	0.059 0.083

Values indicate mean ± SD. The p-value corresponds to the comparison of the two groups using Related-Samples Wilcoxon Signed Rank Test. The table depicts the parametric mean instead of the median in order to denote subtle changes in the scores. Given the use of rank sum tests, medians may be the same although ranks differ, indicated by a significant test result. *indicates significant result at the 5% level, corrected for multiple comparisons (number of tests: 4).

Results indicated CST was more effective than RO&RM in improving attention, with a smaller, less robust effect on episodic memory. This suggests that CST primarily targets attentional processes, which may indirectly support other cognitive functions.

### Executive Functions

CDAD total and subscale scores before and after training are depicted in Table 3. Related-Samples Wilcoxon Signed Rank Test revealed no significant difference between CDAD total score before and after training for either group (all *p* > 0.1). However, effective performance of activities (subscale Executing CDAD) improved after training (*p* = 0.046) in the CST group although the effect did not survive correction for multiple comparisons. When looking at the different classes of activities separately, our analyses revealed CST induced improvements for initiating instrumental activities (*p* = 0.048), planning leisure activities (*p* = 0.008), and executing basic activities (*p* = 0.046) whereas planning basic activities (*p* = 0.009), executing instrumental (*p* < 0.001) and executing leisure activities (*p* = 0.002) further deteriorated after training. However, only the change in executing instrumental activities remained significant after *p*-value correction. No significant changes were observed for other subscales or any subscales in the RO&RM group. Moreover, there were no significant baseline group differences for any of the CDAD scales investigated (all *p* > 0.05). CST showed some potential to improve specific aspects of executive functioning (e.g., initiating and planning certain activities), but these effects were inconsistent and not robust after correcting for multiple comparisons. The deterioration in some areas suggests that CST may not uniformly benefit in all the initiating, planning, and executing aspects of executive function as measured by the CDAD.

**Table 3:** Executive functions before and after training

	*Pre-test*	*Post-test*	*P value*
CST group (*n* = 80)			
CDAD total Initiating Planning Executing	34.55 ± 9.73 11.43 ± 2.64 11.20 ± 3.05 11.90 ± 4.49	34.13 ± 9.06 11.66 ± 2.40 11.20 ± 2.81 11.95 ± 4.43	0.313 0.208 0.650 0.046
Initiating basic activities Initiating instrumental activities Initiating leisure activities	5.91 ± 1.07 4.73 ± 1.14 0.66 ± 0.48	5.86 ± 0.94 4.93 ± 1.13 0.69 ± 0.47	0.440 0.048 0.157
Planning basic activities Planning instrumental activities Planning leisure activities	6.28 ± 1.49 4.46 ± 1.39 0.51 ± 0.50	6.03 ± 1.57 4.24 ± 1.25 0.60 ± 0.49	0.009 0.058 0.008
Executing basic activities Executing instrumental activities Executing leisure activities	5.76 ± 1.38 5.68 ± 2.85 0.51 ± 0.50	5.71 ± 1.44 4.94 ± 2.71 0.39 ± 0.49	0.473 < 0.001* 0.002
RO & RM group (n = 80)			
CDAD total Initiating Planning Executing	34.58 ± 9.68 11.41 ± 2.61 11.25 ± 3.09 11.92 ± 4.46	34.55 ± 9.61 11.40 ± 2.51 11.27 ± 3.07 11.93 ± 4.47	0.461 1.000 0.157 1.000
Initiating basic activities Initiating instrumental activities Initiating leisure activities	5.91 ± 1.07 4.71 ± 1.13 0.66 ± 0.48	5.93 ± 1.05 4.70 ± 1.10 0.68 ± 0.47	0.317 0.564 0.317
Planning basic activities Planning instrumental activities Planning leisure activities	6.28 ± 1.49 4.50 ± 1.41 0.51 ± 0.50	6.28 ± 1.49 4.51 ± 1.40 0.53 ± 0.50	1.000 0.317 0.317
Executing basic activities Executing instrumental activities Executing leisure activities	5.75 ± 1.37 5.69 ± 2.83 0.51 ± 0.50	5.75 ± 1.37 5.69 ± 2.83 0.51 ± 0.50	1.000 1.000 1.000

Values indicate mean ± SD. The p-value corresponds to the comparison of the two groups using Related-Samples Wilcoxon Signed Rank Test. The table depicts the parametric mean instead of the median in order to denote subtle changes in the scores. Given the use of rank sum tests, medians may be the same although ranks differ, indicated by a significant test result. *indicates significant result at the 5% level, corrected for multiple comparisons (number of tests: 26).

### Associations Between Improved Attention and Changes in Executive Functions

**Fig. 1:**
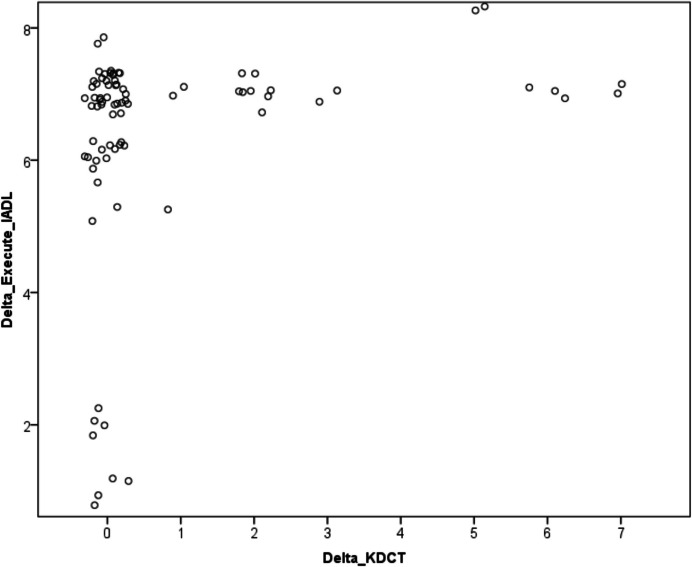
Enhanced attention slows down executive function decline. The scatter plot indicates a hyperbolic relationship between the increase in attention and the decrease in executive function after CST. Figure created using SPSS and the graph command with jittered points to display overlapping data. Post-processing of graph and axis labels using GIMP 2.10.6. IADL, Instrumental Activities of Daly living; KDCT, Kendrick Digit Copying Test

Next, we examined whether changes in executive functions are positively associated with the improvements in attention after CST. In the first step, we performed a Spearman correlation between the change in executing instrumental activities and the change in KDCT scores. This resulted in a significant positive correlation (*ρ* = 0.33, *p* = 0.002) indicating that improvements in attention were associated with lesser executive function decline (see Fig. 1). Conversely, there was no such correlation for the change in KOLT scores (*ρ* = 0.13, *p* = 0.239). Comparing the two correlations using Fisher z-transformation (Lee & Preacher, 2013) revealed a significant difference at *p* = 0.036, indicating that attention improvement but not episodic memory improvement was associated with lesser executive function decline. Ordinal regression analysis confirmed correlation results (−2 Log Likelihood = 28.60, Chi-Square = 20.45, *p* = 0.002) and indicated that executive function decline can be slowed down by improved attention.

Finally, exploratory correlation analysis was performed for the CDAD subscale scores whose changes did not survive p-value correction. This revealed a positive correlation between improvements in attention with the improvement in executing activities (ρ = 0.40, *p* < 0.001), the improvement in initiating instrumental activities (ρ = 0.49, *p* < 0.001) and the improvement in planning leisure activities (ρ = 0.35, *p* = 0.01). However, exploratory analysis also revealed that improvements in episodic memory (KOLT sub-score) were correlated with improvements in initiating instrumental activities (ρ = 0.38, *p* = 0.001) and planning leisure activities (ρ = 0.32, *p* = 0.004). Results indicated that attention improvement appears to play a role in slowing executive function decline, particularly in activities requiring initiation and planning. Episodic memory improvements, while less strongly associated, may also contribute to certain aspects of executive functioning.

## Discussion

Our results indicate that six weeks of daily CST compared to simple RM&RO training can significantly increase attention and, to a lesser extent episodic memory, confirming hypothesis 1. However, we found that overall executive functions did not significantly change, as assessed using the CDAD total score, disproving our hypothesis 2. Instead, executing instrumental activities further deteriorated after training. Moreover, there was a significantly positive correlation between the change in executing instrumental activities and the increase in attention, while regression analysis indicated that improvements in attention were associated with lesser executive function decline, confirming hypotheses 3 and 4. The relationship between attention and executive functions was explored only in the CST group, as this was the only group that demonstrated significant improvements in attention. Including the RO&RM group, which showed no significant change in attention, would have limited the interpretability of the correlation and regression analyses.

### Improved Attention and Episodic Memory After CST

The findings of this investigation underscore the considerable advantages of CST regarding attentional capacities and episodic memory, with enhancements in attention being particularly pronounced. The cohort receiving CST exhibited a statistically significant elevation in KDCT scores, an indicator of attentional performance, which was not mirrored in the RO&RM cohort. Linear regression analysis corroborated that the advancement in attentional abilities was significantly superior in the CST group relative to the RO&RM group. Furthermore, episodic memory, quantified through KOLT scores, demonstrated improvement within the CST group; however, this effect was less pronounced and did not exhibit a significant difference from the RO&RM group. These results align with extant literature illustrating the effectiveness of CST in augmenting attentional and memory functions. For instance, Spector and associates (2003) revealed that CST contributed to gains in cognitive functioning, notably in fields that require sustained concentration and memory access (Spector et al., 2003). In a similar context, Orrell and team (2014) revealed that CST effectively enhanced attentional skills and verbal expression in individuals facing dementia (Orrell et al., 2014). The more pronounced effect on attention in contrast to episodic memory may be indicative of the nature of CST activities, which frequently encompass tasks that require concentration, such as word games, creative endeavors, and discussions (Malhotra, 2018; Orrell et al., 2014). Attention serves as a core cognitive function that supports a range of other processes, including the ability to remember, and boosting it might enhance performance in memory-centric activities (Foldi et al., 2002). Nevertheless, the comparatively diminished impact on episodic memory suggests that CST may benefit from incorporating additional memory-specific training exercises to bolster this particular cognitive domain. Subsequent research could explore the potential benefits of integrating memory training methodologies, such as spaced retrieval or errorless learning, into CST to further enhance outcomes related to episodic memory.

### Executive Functions Before and After the CST

The findings of this inquiry highlight the complicated interactions of CST concerning executive functions in individuals with mild to moderate dementia. Although there was no statistically significant enhancement in the overall CDAD score, particular subscales exhibited noteworthy alterations. For example, the CST cohort evidenced marginal advancements in the effective execution of activities (Executing CDAD subscale), the initiation of instrumental activities, the planning of leisure activities, and the execution of fundamental activities. Nonetheless, these advancements did not withstand adjustments for multiple comparisons, indicating that the effects were relatively modest. In contrast, the planning of basic activities, the execution of instrumental activities, and the execution of leisure activities experienced a deterioration following the intervention, with the decline in executing instrumental activities remaining statistically significant post-correction for p-values. These heterogeneous findings correspond with prior literature, which has indicated that CST can enhance certain dimensions of executive functioning, albeit not uniformly across all domains. For example, Spector et al. (2003) found that CST improved cognitive function and quality of life, but the effects on executive functions were less consistent (Spector et al., 2003). As noted by Woods and others (2012), CST facilitates social communication and cognitive involvement, yet its effectiveness on sophisticated executive functions like organization and problem-solving is rather constrained (Woods et al., 2012). The observed regression in certain executive functions may reflect the progressive trajectory of dementia, wherein specific abilities decline despite therapeutic interventions. Alternatively, it may suggest that CST, while advantageous for attention and memory, may not adequately address the neural networks that underpin executive control. Subsequent research should investigate whether the integration of CST with focused executive function training could produce more substantial and consistent enhancements.

### Associations Between Improved Attention and Changes in Executive Functions

The investigation also examined the association between enhancements in attention and alterations in executive functions subsequent to CST. A notable positive correlation was identified between enhancements in attention (KDCT change scores) and a reduced decline in the execution of instrumental activities, indicating that augmented attention may contribute to the preservation of certain executive functions. Numerous studies validate the association between attention and the management of executive functions. For instance, scholarly works have underscored that attention constitutes a fundamental element of executive functioning, facilitating individuals in maintaining focus, shifting attention, and sustaining cognitive effort during intricate tasks (Desai et al., 2024; Posner et al., 2006). In a related sense, academic work has stressed the relevance of attention management in working memory, which is key for proficient planning and tackling problems (Baddeley, 2003). The exploratory analysis additionally indicated that enhancements in attention were associated with improvements in executing activities, initiating instrumental tasks, and planning leisure pursuits, thereby reinforcing the concept that attention bolsters various facets of executive functioning. Notably, enhancements in episodic memory were also linked to superior performance in initiating instrumental activities and planning leisure pursuits, although these correlations were of a lesser magnitude compared to those related to attention. This observation implies that while attention occupies a pivotal role in the preservation of executive functions, episodic memory may also play a contributory role, albeit to a diminished extent. These findings are congruent with research indicating that cognitive interventions aimed at improving attention can yield cascading advantages for other cognitive domains. For instance, Belleville et al. (2011) found that attention training improved both attentional control and working memory in older adults (Belleville et al., 2011). The present study expands upon this body of literature by illustrating that enhancements in attention may assist in mitigating the decline of executive functions among individuals diagnosed with dementia. Future inquiries should investigate the mechanisms at play in these associations, such as modifications in prefrontal cortex activity or connections, to enrich our understanding of CST's impacts.

By demonstrating that CST can significantly improve attention and episodic memory, and exploring the relationship between attention and executive function changes, this project provides valuable insights that can inform therapeutic interventions. Additionally, it is important to distinguish CST from cognitive training programs. CST is not a structured training regimen targeting specific cognitive domains through repetitive practice. Instead, it is a psychosocial intervention that uses themed group activities to stimulate a broad range of cognitive functions in a socially supportive environment. While CST may lead to improvements in attention and memory, these outcomes emerge through engagement and stimulation rather than formal training.

CST could be adapted to better fit the cultural context of Chinese populations, ensuring that the therapy is both effective and culturally sensitive. This involves adapting activities to align with cultural preferences and values, such as incorporating traditional Chinese music, festivals, and culturally relevant topics into the therapy sessions. Given the collectivist nature of Chinese culture, fostering group cohesion and mutual support during therapy sessions can enhance engagement and effectiveness. Additionally, communication styles in Chinese culture may be more reserved, so CST adaptations might involve encouraging more active participation and expression in a culturally sensitive manner, through structured activities that gradually build confidence (Wong et al., 2018). Key cultural issues identified included less active opinion sharing and a preference for practical activities with recognition (Wong et al., 2018). Another study highlighted the need for modifications in CST to suit the reserved personality traits and potential role conflicts for family caregivers in Chinese populations. This research emphasized the importance of culturally adapting the content and delivery format of CST (Wong et al., 2017). Practical recommendations for enriching CST within Chinese culture include incorporating traditional elements such as calligraphy, tea ceremonies, and discussions about historical figures to make CST sessions more engaging and culturally relevant. Utilizing familiar contexts and objects, such as local news, traditional foods, and common household items, can stimulate memory and cognitive functions. Designing activities that promote social interaction and collective participation can help leverage the collectivist nature of Chinese culture to enhance therapy outcomes. By incorporating these culturally sensitive elements and referencing the supporting literature, the discussion on the use of CST within Chinese culture can be enriched, highlighting its potential benefits and adaptations.

### Limitations

Our study has a number of limitations. First, data were analyzed retrospectively. Second, we failed to document and control for medication intake during study participation. Thus, differences in pharmacological treatments between groups may bias our results. At best, they contribute to an increased variance in measures observed. Additionally, our version of CST was specifically optimized to the needs and idiosyncrasies of participants with dementia in Hong Kong which need to be further validated with the international versions of CST. Essentially, a larger sample size recruitment is needed to confirm the observed effects and address potential variability. A longer follow-up can justify the sustainability of improvements in attention and executive functions. While the potential relevance of executive functions was explored, further study to assess individuals’ ADLs directly should be encouraged. Further study should also be undertaken to explore whether combining CST with other therapies (e.g., executive function training) could yield more robust benefits.

## Conclusion

In this study, we found that CST was effective in improving attention and, to a lesser extent, episodic memory in Chinese older adults with mild to moderate dementia following six weeks of daily intervention. In contrast, RO&RM did not yield significant improvements in these cognitive domains.

This investigation contributes to the growing body of evidence on the differential impacts of CST and RO&RM on cognitive and functional outcomes in dementia care. The demographic comparability of the CST and RO&RM groups—matched in terms of age, gender, education, and baseline cognitive status—supports the internal validity of the findings. The most robust outcome was the improvement in attention within the CST group, which aligns with prior research emphasizing CST’s role in enhancing cognitive engagement and social interaction. Improvements in episodic memory were also observed, though to a lesser degree. However, the effects on executive functioning were mixed. While some gains were noted in areas such as initiating instrumental and planning leisure activities, declines in other domains, including the execution of instrumental tasks, suggest that CST alone may not be sufficient to address the full spectrum of executive dysfunction. Given the modest sample size and variability in these outcomes, conclusions regarding executive function should be interpreted with caution.

Importantly, improvements in attention were significantly associated with reduced decline in certain executive functions, reinforcing the interdependence of these cognitive domains. This finding is consistent with theoretical models that position attention as a foundational process for higher-order cognition (Baddeley, 2003; Posner & Petersen, 1990).

In summary, CST shows promise as a culturally adaptable, non-pharmacological intervention for enhancing attention and memory in older adults with dementia. Future studies should explore the potential benefits of combining CST with targeted executive function training and examine the underlying neural mechanisms to optimize therapeutic outcomes.

## Data Availability

No datasets were generated or analysed during the current study.
